# Brucellosis seroprevalence in cattle in China during 2014–2024: a systematic review and meta-analysis

**DOI:** 10.1080/22221751.2024.2417859

**Published:** 2024-10-25

**Authors:** Zihan Tian, Liyun Wan, Jie Pei, Tingting Li, Xiaozhong Wang, Peng Yuan, Aizhen Guo, Yingyu Chen

**Affiliations:** a National Key Laboratory of Agricultural Microbiology, College of Veterinary Medicine, Huazhong Agricultural University, Wuhan, People’s Republic of China; b Hubei International Scientific and Technological Cooperation Base of Veterinary Epidemiology, The Cooperative Innovation Center for Sustainable Pig Production, Wuhan, People’s Republic of China; c Hubei Provincial Centre for Animal Disease Control and Prevention, Wuhan, People’s Republic of China; d Yichang Animal Disease Prevention and Control Centre, Yichang, People’s Republic of China

**Keywords:** *Brucella*, cattle, prevalence, chin, epidemiology

## Abstract

Brucellosis, caused by several species of *Brucella*, continues to be a significant illness that poses a global threat to public health. China remains a persistent hotspot for brucellosis, despite the implementation of extensive control measures. This study aims to conduct a systematic review and meta-analysis of the seroprevalence of bovine brucellosis in different breeds and regions of China from 2014-2024, and to provide predictions on the future prevalence patterns of brucellosis in cattle and humans. The analysis comprised a total of 80 research studies, which consisted of 187 datasets and a combined sample size of 3,130,706. We estimated the overall pooled seroprevalence of bovine brucellosis in China to be 1.5% (95% CI: 0.6-2.6%). Subgroup analysis revealed that the seroprevalence in dairy cattle was 3.1%, surpassing the seroprevalence in beef cattle (1.3%) and yak (1.5%). Regions that had authorized vaccination programmes exhibited higher seroprevalence (1.8%) compared to regions that did not have vaccination (0.5%). Notably, the study observed a simultaneous rise in both the prevalence of brucellosis in cattle and the number of human brucellosis cases. This suggests that high-quality routine surveillance of brucellosis in cattle will be essential for predicting and responding to cases in humans. Additionally, given the existing prevention and control measures, brucellosis will likely continue to be prevalent in both cattle and people. This systematic review will assist policymakers in adjusting animal surveillance and interregional livestock movement policies, ultimately contributing to the public safety goal of preventing brucellosis in humans by controlling it in animals.

## Introduction

Brucellosis is a highly contagious zoonotic disease that poses substantial risks to both animal and human health. It is caused by various species of the genus *Brucella*, from which, *B. abortus*, *B. melitensis*, and *B. suis* are the most important ones with respect to their zoonotic potential [1].

Approximately, 0.5 million people are infected with brucellosis annually [2]. In China, the incidence of brucellosis in humans had a consistent upward trend from 2004 to 2021, significantly increasing 8.2% [3]. And the main mode of transmission is contact with sick livestock such as sheep, goats, and cattle[4]. Human brucellosis is primarily spread through direct or indirect contact with infected animals, their bodily fluids, or contaminated products like meat, milk, and other dairy products[5]. Direct transmission is especially common during parturition, where exposure to the placenta or aborted fetuses of infected animals poses a significant risk [6].

Bovine brucellosis, primarily caused by *B. abortus*, mainly causes abortion, infertility, and milk reduction in cattle, and leads to major financial losses for farmers through decreased livestock productivity and reproduction [7,8]. It has worldwide geographical distribution. Except very few countries in Western and Northern Europe, Canada, Japan, Australia, and New Zealand are believed to be free from the agent, most other countries, especially developing countries, have very high incidences, such as the Middle East, the Mediterranean region, sub-Saharan Africa, India, Peru, Mexico, and China [9]. In district Multan, Pakistan, the overall seroprevalence rate was 12.65% in cattle, 80% of the positive cattle were identified as *B. abortus*, 20% were *B. melitensis* by PCR [10]. In Kajiado County of Kenya, the seroprevalence of bovine brucellosis was 18.35%, with a herd prevalence of 67.65% [11]. In Brazil, the highest seroprevalence was 11% in Acre, with a lowest of 0.4% in the Federal District [12].

In China, as early as 2012, the China Ministry of Agriculture and Rural Afairs classified brucellosis as one of the 16 infectious diseases to be controlled and eliminated in its Medium and Long-Term National Plan for Prevention and Control of Animal Diseases (2012–2020) (http://www.moa.gov.cn/xw/zwdt/201205/t20120530_2678977.htm). In 2022, Chinese government launched the “Five-Year Action Plan for Brucellosis Prevention and Control in Livestock (2022–2026).” During the controlling, the Rose Bengal Plate Test (RBPT), Milk Ring Test (MRT), Standard Agglutination Test (SAT), Complement Fixation Test (CFT), indirect ELISA (iELISA), competitive ELISA (cELISA) and PCR were used for the diagnosis according to the national standard (GB/T 18646-2018) in China. And the vaccines currently approved by the Chinese government for cattle include *B abortus* A19 strain, *B. suis* S2 strain, and *B. melitensis* M5 strain. But as brucellosis has different immune strategies in different regions in China, and the lack of effective differential diagnostic methods has become a bottleneck in brucellosis diagnosis, which to some extent interferes with the accuracy of the test prevalence [13].

A systematic review and meta-analysis revealed that the overall seroprevalence of brucellosis in dairy cattle herds was 1.9% during 2008-2018, and indicated an upward trajectory in the animal-level prevalence, rising from 1.6% in 2008–2012 to 2.6% in 2013–2018 [14,15]. However, after 2018, epidemiological data on the prevalence of bovine brucellosis in China has been limited.

Comprehensive epidemiological data, including the prevalence of different cattle breeds and production purposes, as well as the temporal and spatial distribution of the disease, are essential prerequisites for implementation a comprehensive campaign to prevent and control bovine brucellosis in China. Due to the lack of recent data on cattle brucellosis, especially beef cattle data over the past decade, we conducted this study, aimed at investigating the pooled seroprevalence of bovine brucellosis among various cattle breeds, including dairy, beef, and yak. Additionally, the study explored the influence of areas implementing different policies on the seroprevalence estimates by cattle breed, as well as the trends of brucellosis prevalence in both humans and cattle.

The findings from this analysis provide valuable insights into the epidemiology of bovine brucellosis and highlight the importance of considering breed-specific differences when designing and implementing disease control programmes in China and other endemic regions. Understanding the variations in seroprevalence across different cattle breeds and areas is crucial for tailored interventions to the local context. Furthermore, monitoring the trends of brucellosis prevalence in both human and animal populations can help identify any emerging patterns that may require adjustments to the control programme over time.

## Methods

### Search strategy

This systematic review and meta-analysis was conducted in accordance with the Preferred Reporting Items for Systematic Reviews and Meta-analyses (PRISMA) reporting guideline for the design and analysis of selected qualified studies [16].

A literature search was conducted to identify articles published between January 1, 2014, and June 1, 2024. The goal was to gather a comprehensive collection of articles on the seroprevalence of bovine brucellosis in China, published in both Chinese and English. Articles were obtained from five databases, including China National Knowledge Infrastructure (CNKI), VIP Data, PubMed, and Google Scholar. The article search utilized the Medical Subject Headings (MeSH) terms, employing Boolean operators “AND” “OR,” and “NOT” to connect the MeSH terms. We combined searches for brucellosis (*Brucella*, *B. abortus*), cattle (bovine, yak), prevalence (seroprevalence, epidemiology), and China (excluding this MeSH term in Chinese databases to avoid limiting the search). We have also excluded articles that pertain to meta-analysis (systematic review). Within the Chinese databases, we utilized functions such as synonym expansion and fuzzy search to enhance our search capabilities.

### Eligibility criteria and literature review

The following criteria were established for inclusion in the study: (1) The study conducted a cross-sectional survey to determine prevalence; (2) The study presented the total number of samples tested and the number of positive results.; (3) The study mentioned specific testing methods; (4) The samples were taken from individual cattle and were not combined; (5) The sample size was larger than 30; (6) Random sampling was employed.

Only those articles that satisfied the specified criteria were subjected to further analysis. Additionally, we did not reach out to the original authors for further details, did not obtain articles that were inaccessible, and did not incorporate unpublished grey research.

This analysis focused exclusively on the seroprevalence of brucellosis in cattle, deliberately excluding pathogen prevalence data determined by PCR. This because (1) very few publications were focused on PCR for cattle brucellosis, (2) PCR was used for the confirming of brucellosis in serological positive individual/herds in all the published research, not for the random selection, which introduced sample bias.

### Quality assessment and data extraction

Data extracted from each identified study included the score, the first author, the province surveyed, the survey year, the detection method, the cattle breed, the sample size, and the number of positive samples.

The Joanna Briggs Institute (JBI) quality appraisal checklist was used to assess the quality of individual papers, which was utilized for studies reporting prevalence data published [17,18]. Based on the nine questions from the JBI checklist, five key items were identified for further: the appropriateness of the sampling frame, adequacy of the sample size, clarity in describing the study subjects, specification of the survey years, and reliability of the measurement methods. Each aspect was given a score of 1. Studies scoring 5 points were considered high quality, 3–4 points as moderate quality, and 1–2 points as low quality. Articles scoring 0 points were excluded.

Two investigators (ZT and TL) independently extracted data and assessed the quality of the publication before discussing the results together. All discrepancies were resolved by YC.

### Statistical analysis

Data extraction and initial sorting were performed using Microsoft Excel. The data analysis was conducted using STATA 17.0 (Stata Corporation, College Station, TX, USA). The pooled seroprevalence of brucellosis in cattle was estimated, and the test for heterogeneity between the combined study results was carried out. Due to the presence of outliers (**0**) in the data, the Bartlett correction and Freeman-Tukey double arcsine transformation methods were separately employed to preprocess the proportional data [19,20]. This allowed us to obtain the prevalence estimates (95% CI) and sampling standard error for each study. The heterogeneity test revealed I² > 75%, indicating substantial heterogeneity; thus, the random effects model (REM) was used for pooled estimates and subgroup analyses. The heterogeneity results were similar between the two data correction methods (I² = 100% and 99.979%). Given the non-normal distribution of the transformed data, we proceeded with the meta-analysis of proportions using the Freeman-Tukey double arcsine transformed proportions.

Potential sources of heterogeneity were further explored through subgroup analysis and meta-regression analysis. Sensitivity analysis and publication bias tests were conducted to ensure the robustness and reliability of the study findings. The meta-analysis was conducted in accordance with the PRISMA guidelines [16,21].

Time series analysis was conducted using the AutoRegressive Integrated Moving Average (ARIMA) model in IBM SPSS Statistics, version 24.0. The ARIMA model parameters (p, d, q) were determined through the analysis of autocorrelation function (ACF) and partial autocorrelation function (PACF) plots. The models were then fitted and validated through residual analysis to ensure the residuals were consistent with white noise. Forecasts were generated from the validated models to predict trends in bovine brucellosis seroprevalence from 2023 to 2027 and human brucellosis cases from June 2024 to December 2027. The forecasted values were utilized to guide potential public health interventions.

### Publication bias and sensitivity analysis

When conducting publication bias tests, the results indicated an asymmetric funnel plot and a statistically significant Egger's test (*P* < 0.05), suggesting the presence of publication bias (Figure S1). Given that the data were not normally distributed and involved single-group proportion analysis, it is evident that the results were more descriptive in nature rather than indicative of robust statistical significance. Considering this, it was deemed preferable to explore these biases by elucidating the sources of heterogeneity rather than excluding studies that exhibited bias. Additionally, the effect sizes obtained from the double arcsine transformation cannot be used for sensitivity analysis using the “metaninf” function, as this function is designed for raw proportions.

## Result

### Characteristics of included studies

**Figure 1.**
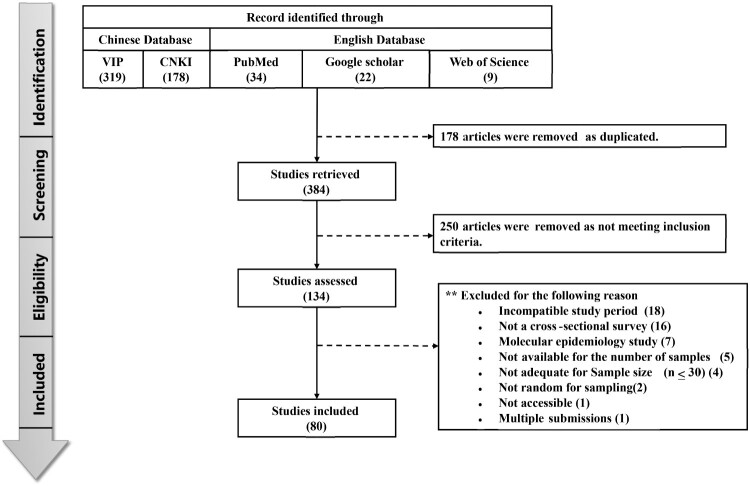
PRISMA flow diagram for systematic review depicting phases of identification of studies.

The initial database search using the keywords described above identified a total of 562 articles related to brucellosis infection in cattle in China. Based on the predefined inclusion criteria and a quantitative assessment, 80 of these articles were selected for inclusion in the meta-analysis (Figure 1). Of the 80 included articles, 14 were scored as high quality, 48 were scored as medium quality, and 18 were classified as low quality (Table S1).

### Overall estimation

**Figure 2.**
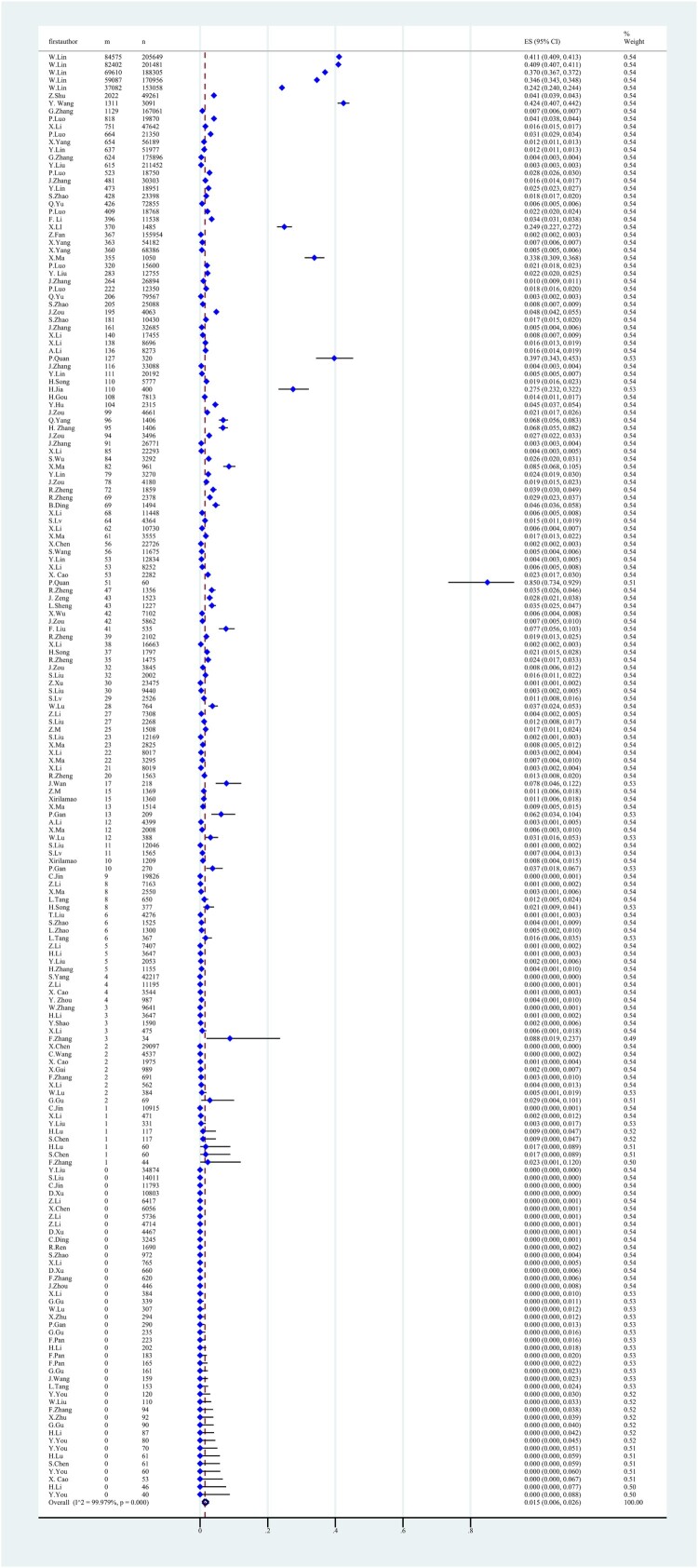
Forest plot of brucellosis seroprevalence in cattle in China. The data were weighted using a random effects model. Each blue square represents the effect size (ES) for individual studies, the horizontal black lines indicating the 95% confidence interval (CI). The vertical dashed line indicates the overall pooled effect size, and the diamond at the bottom represents the overall summary effect. “m” represents the total number of samples, “n” represents the number of positive samples, and “ES” represents to the seroprevalence of brucellosis in cattle in each study.

From 2014 to 2024, a total of 80 studies comprising 187 datasets and 3,130,706 field samples were analysed. The individual dataset prevalence estimates ranged from 0% to 85%. The national-level pooled prevalence estimate was 1.5% (95% CI: 0.6-2.6%) (Figure 2).

### Pooled bovine brucellosis seroprevalence across the administrative districts or provinces of China

**Figure 3.**
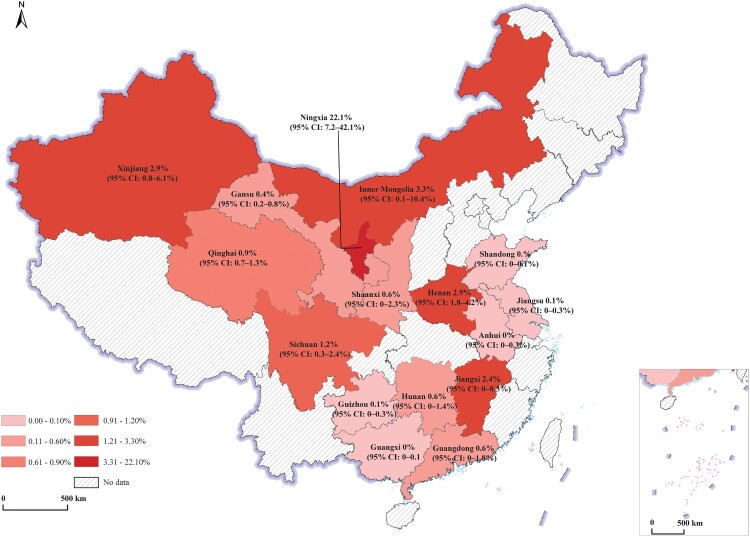
Map of seroprevalence of bovine brucellosis in China. The map was created using a standard map from the website of the Ministry of Natural Resources of China, with drawing check No.: GS(2023)2764. The base map has not been modified. The inset depicts the territorial waters of the South China Sea.The map displays the seroprevalence rates (with 95% confidence intervals) in various provinces, using a colour gradient to indicate different prevalence levels. Darker red shading corresponds to higher seroprevalence, ranging from 0.01% to 22.1%. Provinces without available data are represented with diagonal hatching.

The analysis of pooled seroprevalence across China’s administrative districts revealed significant regional differences in bovine brucellosis prevalence. Ningxia had the highest seroprevalence at 22.1%, followed by Inner Mongolia, Xinjiang, and Henan, each with rates around 3%. Other provinces generally exhibited lower seroprevalence, with many reporting rates below 1%. These findings highlight Ningxia as a particularly high-risk area, while most other regions show comparatively low levels of seroprevalence. Provinces with fewer than one dataset were excluded from the analysis due to a lack of representative data (Figure 3).

According to the notice from the Animal Husbandry and Veterinary Bureau of the Ministry of Agriculture and Rural Affairs regarding the list of counties and dairy farms eligible for mandatory *Brucella* vaccination in 2024 (http://www.xmsyj.moa.gov.cn/gzdt/202402/t20240222_6448891.htm), the vaccination programme has been implemented across 17 provincial-level administrative regions, including Beijing, Tianjin, Hebei, Shanxi, Inner Mongolia, Liaoning, Jilin, Heilongjiang, Shandong, Henan, Sichuan, Tibet, Shaanxi, Gansu, Qinghai, Ningxia, and Xinjiang. These provinces have been classified as high-baseline areas for brucellosis, where the human incidence rate exceeds 1/100,000, or where the number of counties with uncontrolled outbreaks in livestock accounts for 30% of the total counties. As a result, these provinces implement mandatory vaccination policies. In comparison, the remaining provinces were designated as low-baseline areas on the epidemiological surveillance data, primarily adopting strategies focused on monitoring, eradication, and risk prevention. The seroprevalence of brucellosis was estimated to be 1.8% (95% CI: 0.8–3.3%) in high-baseline area, while it was 0.5% (95% CI: 0.2–0.9%) in low-baseline area (Table 1). The observed seroprevalence aligns with the categorization of provinces.

**Table 1.** Sub-group analysis of the seroprevalence of bovine Brucellosis.

Variation	Sub-variation	No. dataset	No. tested	No. positive	(%) Overall (95%CI)	(%) I2
Area	High-baseline	141	2,752,148	350,437	1.8 (0.8-3.3)	99.987
	Low-baseline	46	378,558	2,491	0.5 (0.2-0.9)	99.374
Breed	Diary cattle	55	1,489,933	338,153	3.1 (0.5-7.7)	99.994
	Beef cattle	19	158,166	1,581	1.3 (0.7-2.1)	99.158
	Yak	4	11,013	165	1.5 (0.7-2.6)	89.734
Survey year	2014-2018	76	1,136,589	503,371	1.2 (0.6-2.0)	99.907
	2019-2024	105	1,591,298	301,397	1.4 (0.2-3.6)	99.987

Note: I² indicates the heterogeneity across datasets, with values close to 100% indicating high heterogeneity.

### Pooled bovine brucellosis seroprevalence in China during the selected periods

**Figure 4.**
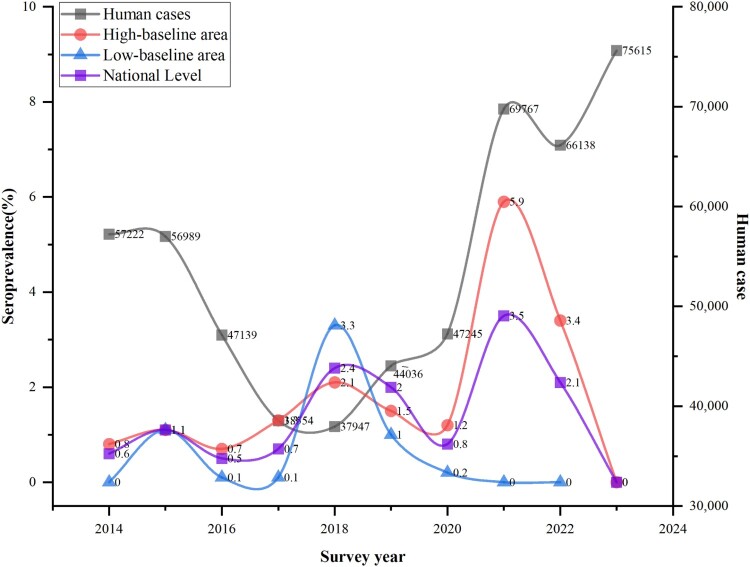
Trends of bovine brucellosis seroprevalence and human brucellosis cases in China (2014-2024). The graph uses dual Y-axes, with the left Y-axis representing bovine brucellosis seroprevalence (%) and the right Y-axis representing reported human brucellosis cases. The purple-square-line represents the national bovine brucellosis seroprevalence, the red-circle-line shows the seroprevalence in high-baseline areas, the blue-triangle-line indicates the seroprevalence in low-baseline areas, and the graysquare-line represents the number of reported human brucellosis cases.

The national-level bovine brucellosis prevalence exhibited a wave-like pattern over the study period. Beginning at 0.6% in 2014, the prevalence increased to 1.1% in 2015 before declining to 0.5% in 2016. Thereafter, the national cattle brucellosis prevalence fluctuated between 0.7% and 2.4% from 2017 to 2020, reaching a peak of 3.5% in 2018. In 2021 and 2022, the national prevalence was recorded at 2.1% and 2.0%, respectively. Notably, the 2023 data showed a prevalence of 0%. A more nuanced regional analysis highlighted significant disparities in cattle brucellosis epidemiology. In the high-baseline area, the brucellosis prevalence followed a similar wave-like pattern, with values of 0.8%, 1.1%, 0.7%, 1.3%, 2.1%, 1.5%, 1.2%, 5.9%, 3.4% and 0, observed from 2014 to 2023, with no data available for 2024. In contrast, the low-baseline area exhibited lower and more variable prevalence, ranging from 0% to 3.3% between 2014 and 2022, with no data available for 2023 and 2024 (Figure 4).

Paralleling the trends in bovine brucellosis seroprevalence, the total number of reported human brucellosis cases in China exhibited an overall increasing trajectory over the 2014–2024 study period (https://www.ndcpa.gov.cn/jbkzzx/c100016/common/list.html). From a baseline of 57,222 cases in 2014, the annual incidence decreased each year, reaching a low point of 37,947 cases in 2018. However, this downward trend was followed by a rise to 44,036 cases in 2019 and 47,245 cases in 2020, 69,767 in 2021, and 66,138 in 2022. The peak was reached in 2023, with 75,615 reported human brucellosis cases (Figure 4).

**Figure 5.**
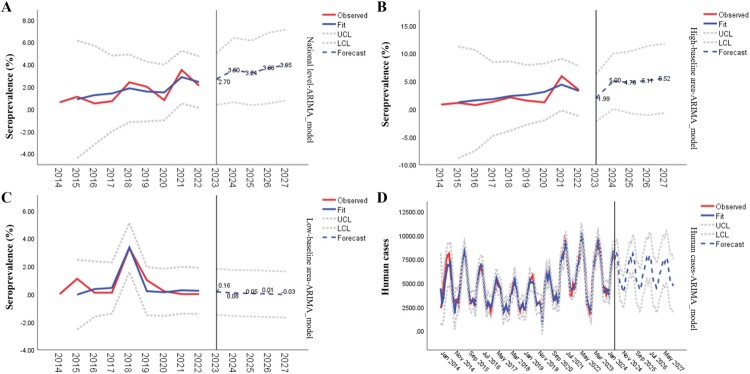
Prediction of the seroprevalence of brucellosis in cattle and human case numbers based on time series models. ARIMA model prediction (2014-2027) of A: bovine brucellosis seroprevalence at national level. B: bovine brucellosis seroprevalence in high-baseline area, C: bovine brucellosis seroprevalence in low-baseline area. D: Human brucellosis cases in China. Abbreviations: Fit (The line that represents the fitted model to the observed data), UCL (Upper Control Limit), LC (Lower Control Limit).

According to the time series forecast, the ARIMA model at the national level reveals a small rise in bovine seroprevalence, from 2.70% to 3.95% (Figure 5A). This suggests the importance of continued vigilance and potential interventions to manage brucellosis on a national scale. The model of high-baseline area with a significant increase in seroprevalence from 1.99% to 5.52% (Figure 5B), indicating the need for additional strategies to address this potential challenge. In the low-baseline area, the results indicated a consistent pattern with slight variations and forecasted values near 0% (Figure 5C), validating the efforts to establish brucellosis-free zones.

The ARIMA model forecast for human cases from June 2024 to December 2027 indicates intermittent fluctuations, with the overall number of occurrences oscillating between approximately 2,500 and 12,500 (Figure 5D, Table S4). This trend emphasizes the importance of ongoing monitoring and efficient public health efforts in managing and reducing the impact of brucellosis on human populations.

### Pooled seroprevalence of bovine brucellosis by cattle breed

To account for potential variations in pathogen exposure across different cattle breeds, subgroup analyses were conducted. These variations are influenced by factors such as production purpose, rearing environment, growth cycle, and vaccination status. The study compiled a total of 78 datasets on brucellosis seroprevalence, including 55 for dairy cattle, 19 for beef cattle, and 4 for yaks.

Based on the subgroup analysis, dairy cattle exhibited the highest pooled seroprevalence at 3.1% (95% CI: 0.5–7.7%). Yaks followed with a seroprevalence of 1.5% (95% CI: 0.7–2.6%), while beef cattle had a lower seroprevalence of 1.3% (95% CI: 0.7–2.1%) (Table 1).

Next, we performed a stratified analysis by area and breed. In the high-baseline area, 61 datasets were included. The pooled seroprevalence was estimated to be 3.7% (95% CI: 0.5-9.3%) for dairy cattle and 1.6% (95% CI: 0.8–2.5%) for beef cattle. In the low-baseline area, a total of 13 datasets were included, yielding pooled seroprevalence estimates of 1.1% (95% CI: 0–3.6%) for dairy cattle and 0% (95% CI: 0–0.4%) for beef cattle (Table 2). The yak datasets were exclusively obtained from the high-baseline area, precluding their inclusion in the stratified analysis.

**Table 2.** Seroprevalence of bovine Brucellosis by breed, area, and survey year in China (2014–2024).

Var2		Area		(%)Overall (95%CI)
Var1		High-baseline	Low-baseline	
Breed	Diary cattle	$\frac{3.7\text{\%}\;(0.5\text{-}9.3\text{\%})}{45}$	$\frac{1.1\text{\%}\;(0.0\text{-}3.6\text{\%})}{10}$	33.1% (0.5–7.7%)
	Beef cattle	$\frac{1.6\text{\%}\;(0.8\text{-}2.5\text{\%})}{16}$	$\frac{0.0\text{\%}\;(0.0\text{-}0.4\text{\%})}{3}$	1.3% (0.7–2.1%)
Survey year	2014	$\frac{0.8\text{\%}(0.2\text{-}1.9\text{\%})}{9}$	$\frac{0.0\text{\%}(0.0\text{-}1.9\text{\%})}{2}$	0.6% (0.1–1.6%)
	2015	$\frac{1.1\text{\%}(0.6\text{-}1.7\text{\%})}{22}$	$\frac{1.1\text{\%}(0.0\text{-}3.3\text{\%})}{4}$	1.1% (0.7–1.7%)
	2016	$\frac{0.7\text{\%}(0.0\text{-}1.7\text{\%})}{9}$	$\frac{0.1\text{\%}(0.0\text{-}0.7\text{\%})}{5}$	0.5% (0.1–1.3%)
	2017	$\frac{1.3\text{\%}(0.5\text{-}2.2\text{\%})}{14}$	$\frac{0.1\text{\%}(0.0\text{-}0.4\text{\%})}{8}$	0.7% (0.3–1.3%)
	2018	$\frac{2.1\text{\%}(0.0\text{-}6.6\text{\%})}{20}$	$\frac{3.3\text{\%}(0.0\text{-}12.7\text{\%})}{6}$	2.4% (0.2–6.3%)
	2019	$\frac{1.5\text{\%}(0.0\text{-}7.2\text{\%})}{19}$	$\frac{1.0\text{\%}(0.0\text{-}2.9\text{\%})}{5}$	2.0% (0.0–6.9%)
	2020	$\frac{1.2\text{\%}(0.0\text{-}10.9\text{\%})}{12}$	$\frac{0.2\text{\%}(0.0\text{-}1.1\text{\%})}{7}$	0.8% (0.0–7.1%)
	2021	$\frac{5.9\text{\%}(0.0\text{-}35.8\text{\%})}{13}$	$\frac{0.0\text{\%}(0.0\text{-}0.7\text{\%})}{3}$	3.5% (0.0–24.3%)
	2022	$\frac{3.4\text{\%}(0.0\text{-}17.7\text{\%})}{9}$	$\frac{0.0\text{\%}(0.0\text{-}0.1\text{\%})}{5}$	2.1% (0.0–12.5%)
	2023	$\frac{0.0\text{\%}(0.0\text{-}0.0\text{\%})}{2}$	No date	0.00% (0.0–0.0%)
	2024	No date	No date	No date

Note:**indicates the number of datasets.

### The correlation between cattle production and brucellosis

Data from the National Bureau of Statistics of China to examine the annual average scale of the cattle industry in each province from 2014 to 2023, including annual cattle numbers, beef and milk production. We further explored potential correlations with bovine brucellosis prevalence using Spearman’s rank correlation coefficient. No significant statistical relationships were found according to the data (Table S2).

### Meta-regression analysis of related subgroups

The meta-regression analysis encompassed 187 observations and utilized REML estimation to quantify the between-study variance (tau² = 0.008273). The residual heterogeneity was substantial (I-squared = 99.979%), with only 0.52% of the between-study variance explained by the covariates (Adj R-squared = 0.52%). This suggests that a considerable portion of the variation remained unexplained. The joint test for all covariates was not statistically significant (F (3,183) = 1.18, *p* = 0.3194) with the Knapp-Hartung modification.

Among the covariates examined, none showed a statistically significant impact on the prevalence of brucellosis in cattle for the model (*p* > 0.05 for all covariates), This suggests that the lack of detailed background information, such as the immune status, age groups, and gender of the sampled cattle, limits a comprehensive understanding and interpretation (Table S3).

## Discussion

*Brucella* infections have significant negative impacts on both animal and human health, leading to severe economic losses and public health threats[22–24]. In addition to causing reproductive failure, which is a hallmark of brucellosis, symptoms in both humans and animals can range from flu-like illness to more severe complications involving multiple organs [6,8]. Despite extensive intervention and control strategies, *Brucella* remains prevalent in China [25]. This comprehensive epidemiological study provides important insights into the prevalence of bovine brucellosis in China, offering a crucial foundation for enhancing disease prevention and control efforts. The key findings highlight the significant variations in seroprevalence across different cattle breeds and the impact of policies implemented in different baseline areas on the observed rates.

The overall pooled seroprevalence of bovine brucellosis in China was estimated at 1.5%, which is concerning given the severe public health implications of this zoonotic disease. The higher seroprevalence observed in dairy cattle (3.1%) compared to beef cattle (1.3%) and yak (1.5%) underscores the need for breed-specific control strategies and suggests that dairy production systems may harbour a greater risk of brucellosis transmission. This could be attributed to factors such as more intensive management practices, increased animal-to-animal contact, and potential exposure to contaminated environments in dairy farms. This finding is consistent with previous studies that have reported higher brucellosis prevalence in dairy herds compared to other cattle production systems [26–28].

Through an analysis of prevalence rates in different baseline areas categorized by whether local policies permit vaccination, we found that regions with authorized vaccination campaigns exhibited higher seroprevalence rates (1.8%) compared to those without vaccination (0.5%). Therefore, adjusting vaccination strategies based on local epidemiological conditions, establishing traceable immunization records, and conducting regular disease screening – especially strengthening the regulation of interregional livestock movement, which has been well implemented for pigs but still has significant gaps for cattle – are crucial steps moving forward.

Notably, the study highlights a clear trend of synchronous increase in the seroprevalence of bovine brucellosis and the number of human brucellosis cases in high-baseline area. Even in low-baseline area, there is a correlation between bovine brucellosis seroprevalence and human cases during certain periods. Moreover, the predictive value and correlation between the seroprevalence of bovine brucellosis and the number of human brucellosis cases suggest that the disease will persist among cattle and humans, with an upward trend observed in cattle. As an ancient zoonotic infectious disease, farm animals are the primary reservoirs of *Brucella*[29]. Previous studies have suggested a strong correlation between the seropositivity rates in humans and animals [30]. Given that brucellosis is strongly associated with occupational exposure [31–33], the simultaneous rise in the prevalence of brucellosis in cattle and the number of human brucellosis cases is a significant concern. Unfortunately, we were unable to obtain occupational distribution data for these cases, which would have added further significance in public health to this finding. These findings underscore the critical need to strengthen surveillance systems and foster collaboration between veterinary and public health authorities to achieve the goal of “One Health” by controlling animal diseases to prevent human cases.

The analysis presented here has several limitations. First, the quality of the included studies varies, and some may have selection bias and information bias. Additionally, most studies lack detailed background information, such as the immunization status, age groups, and gender of the sampled cattle, which limits a comprehensive understanding and interpretation of the results. While we attempted to account for biases and heterogeneity in the data, only 0.52% of the between-study variance was explained by the covariates, indicating that there may still be substantial uncontrolled confounding factors.

Second, in this study, almost all the included studies used serum (only one study used whey) to measure *Brucella* antibodies in cattle. Current serological tests for brucellosis, including RBPT, ELISA, and SAT, are not applicable to DIVA [13,34]. Furthermore, many of the study regions enforce the mandatory use of brucellosis vaccines, and instances of unauthorized vaccine administration even in areas where it is banned have been reported. This can interfere with test results and potentially lead to an higher detection rate [35,36]. Despite these limitations, we adhered strictly to the standard of random sampling and did not exclude such “anomalous” data, as we believe these data represent a complex, cross-sectional scenario influenced by multiple factors and therefore should be retained.

Additionally, despite animal brucellosis being a notifiable disease in some countries within the region, the reported cases often underestimate the true number of positive cases [37–39]. Most of the data included in the analysis were provided by official government veterinarians. However, there are concerns regarding the quantity and quality of studies reported in certain provinces, including publication delays, outdated reporting, and varying research quality. Additionally, in some cases, this may lead to the publication of inaccurate information to avoid accountability. There is even the possibility that false information may be published in some cases to avoid accountability. These issues can result in data that do not accurately reflect the actual situation, hindering timely awareness of recent developments. Ultimately, this can adversely impact the ability of senior policymakers to make informed decisions. Unfortunately, this issue of incomplete reporting and limited accessibility of data is widespread globally [12,39,40].

Lastly, despite the extensive use of MeSH-based searches, it is possible that not all publications related to cattle brucellosis were captured in the selected databases. Some articles may have used specific regional names or abbreviations, potentially leading to the omission of certain relevant data.

## Conclusion

This meta-analysis of 187 datasets on bovine brucellosis seroprevalence revealed notable differences across cattle breeds, with dairy cattle exhibiting the highest pooled seroprevalence at 3.1%, followed by yaks at 1.5% and beef cattle at 1.3%. Stratifying by baseline prevalence areas showed higher seroprevalence in dairy (3.7%) and beef (1.6%) cattle from high-baseline area compared to low-baseline area. However, the meta-regression analysis, including 187 observations, indicated substantial residual heterogeneity (I-squared = 99.979%) that could not be adequately explained by the examined covariates, which accounted for only 0.52% of the between-study variance. These findings suggest that the variations in brucellosis seroprevalence are influenced by a complex interplay of factors, and the lack of detailed information on potential confounding variables limited a comprehensive understanding of the observed epidemiological patterns, highlighting the need for further research to elucidate the drivers of brucellosis transmission and prevalence in diverse cattle populations to inform targeted prevention and control strategies.

## Author contributions

ZT YC and AG conceived the study and secured the funding. ZT, TL and YC collected the publications, extracted the data and assessed the literature quality together. JP, TL, and PY provided help in the study design, ZT, LW, YC, PJ and XW contributed to the results interpretation and discussion. ZT and LW analysed the data and wrote the manuscript. All authors contributed to the manuscript editing and approved the final manuscript.

## Statement of ethics

An ethics statement is not applicable because this study is based exclusively on published literature.

## Acknowledgements

The authors would like to thank Professor Guo, Associate Professor Chen, Dr. Pei and Dr. Li for their technical support.

## Supplementary Material

Supplement_clean.pdf

## References

[1] Laine CG, Johnson VE, Scott HM, et al. Global estimate of human brucellosis incidence. Emerg Infect Dis. 2023;29(9):1789–1797. 37610167 10.3201/eid2909.230052 PMC10461652

[2] Samadi A, Amiri M, Hailat N. The reasons behind long-term endemicity of brucellosis in Low and middle-income countries: challenges and future perspectives. Curr Microbiol. 2024 2024;81(3):82. 10.1007/s00284-023-03605-5 38289422

[3] Wen X, Wang Y, Shao Z. The spatiotemporal trend of human brucellosis in China and driving factors using interpretability analysis. Sci Rep. 2024;14(1):4880. 38418566 10.1038/s41598-024-55034-4 PMC10901783

[4] Tao Z, Chen Q, Chen Y, et al. Epidemiological characteristics of human brucellosis- China, 2016-2019. China CDC Wkly. 2021;3(6):114–119. 34595016 10.46234/ccdcw2021.030 PMC8393115

[5] CfD C. Prevention. estimates human Brucella infections could be four times higher than previously thought. Food Safety. 2023.

[6] Qureshi KA, Parvez A, Fahmy NA, et al. Brucellosis: epidemiology, pathogenesis, diagnosis and treatment–a comprehensive review. Ann Med. 2023;55(2):2295398. 38165919 10.1080/07853890.2023.2295398 PMC10769134

[7] Abdisa T. Review on the reproductive health problem of dairy cattle. J Dairy and Vet Sci. 2018;5(1):1–12.

[8] Pinn-Woodcock T, Frye E, Guarino C, et al. A one-health review on brucellosis in the United States. J Am Vet Med Assoc. 2023;261(4):451–462. 36862545 10.2460/javma.23.01.0033

[9] Liu Z, Gao L, Wang M, et al. Long ignored but making a comeback: a worldwide epidemiological evolution of human brucellosis. Emerg Microbes Infect. 2024;13(1):2290839. 38039063 10.1080/22221751.2023.2290839 PMC10878345

[10] [10] Awais MM, Khadim G, Akhtar M, et al. A study on the epidemiology of brucellosis in bovine population of peri-urban and rural areas of district Multan, southern Punjab, Pakistan. BMC Vet Res. 2024;20(1):39. 38297263 10.1186/s12917-024-03880-9 PMC10832158

[11] Odongo MO, Bebora LC, Gathumbi JK, et al. Seroprevalence and spatial distribution of livestock brucellosis using three serological tests in Kajiado County, Kenya. Open Vet J. 2023;13(12):1583–1596. 38292705 10.5455/OVJ.2023.v13.i12.8 PMC10824078

[12] Dos Santos Rocha ID, Clementino IJ, Canuto de Sousa DL, et al. Distribution, seroprevalence and risk factors for bovine brucellosis in Brazil: Official data, systematic review and meta-analysis. Rev Argent Microbiol. 2024;56(2):153–164. 38177023 10.1016/j.ram.2023.08.002

[13] Cao X, Li S, Li Z, et al. Enzootic situation and molecular epidemiology of Brucella in livestock from 2011 to 2015 in Qingyang, China. Emerging Microbes Infect. 2018;7(1):1–8. 10.1038/s41426-018-0060-y PMC5882930 29615607

[14] Wang Y, Vallée E, Compton C, et al. A novel Bayesian Latent Class Model (BLCM) evaluates multiple continuous and binary tests: A case study for Brucella abortus in dairy cattle. Prev Vet Med. 2024;224:106115. 38219433 10.1016/j.prevetmed.2024.106115

[15] Ran X, Cheng J, Wang M, et al. Brucellosis seroprevalence in dairy cattle in China during 2008–2018: A systematic review and meta-analysis. Acta Trop. 2019;189:117–123. 30308207 10.1016/j.actatropica.2018.10.002

[16] Page MJ, McKenzie JE, Bossuyt PM, et al. The PRISMA 2020 statement: an updated guideline for reporting systematic reviews. Br Med J 2021;29:372:n71. 10.1136/bmj.n71 PMC8005924 33782057

[17] Porritt K, Gomersall J, Lockwood C. JBI's systematic reviews: study selection and critical appraisal. AJN The American Journal of Nursing. 2014;114(6):47–52. 10.1097/01.NAJ.0000450430.97383.64 24869584

[18] Munn Z, Moola S, Lisy K, et al. Methodological guidance for systematic reviews of observational epidemiological studies reporting prevalence and cumulative incidence data. Int J Evid Based Healthc. 2015;13(3):147–153. 26317388 10.1097/XEB.0000000000000054

[19] Barendregt JJ, Doi SA, Lee YY, et al. Meta-analysis of prevalence. J Epidemiol Community Health. 2013;67(11):974–978. 23963506 10.1136/jech-2013-203104

[20] Noma H. Confidence intervals for a random-effects meta-analysis based on Bartlett-type corrections. Stat Med. 2011;30(28):3304–3312. 21964669 10.1002/sim.4350

[21] Sarkis-Onofre R, Catalá-López F, Aromataris E, et al. How to properly use the PRISMA statement. Syst Rev. 2021;10:1–3. 33875004 10.1186/s13643-021-01671-z PMC8056687

[22] Singh BB, Dhand NK, Gill JP. Economic losses occurring due to brucellosis in Indian livestock populations. Prev Vet Med. 2015;119(3-4):211–215. 25835775 10.1016/j.prevetmed.2015.03.013

[23] Angara T, Ismail A, Ibrahim A, et al. Assessment of the economic losses due to bovine brucellosis in Khartoum state. Sudan. International Journal of Technical Research and Applications. 2016;4(2):85–90.

[24] Dadar M, Tiwari R, Sharun K, et al. Importance of brucellosis control programs of livestock on the improvement of one health. Vet Q. 2021;41(1):137–151. 33618618 10.1080/01652176.2021.1894501 PMC7946044

[25] Yang C, Gao J, Xian R, et al. Molecular epidemiology of Brucella abortus isolated from the environment in ningxia Hui autonomous region, China. Infect Genet Evol. 2024;123:105635. 38969194 10.1016/j.meegid.2024.105635

[26] Tulu D. Bovine Brucellosis: Epidemiology, Public Health Implications. And status of Brucellosis in Ethiopia. Vet Med (Auckl). 2022;13:21–30. 35028300 10.2147/VMRR.S347337 PMC8752066

[27] de Alencar Mota ALA, Ferreira F, Ferreira Neto JS, et al. Large-scale study of herd-level risk factors for bovine brucellosis in Brazil. Acta Trop. 2016;164:226–232. 27664333 10.1016/j.actatropica.2016.09.016

[28] Chand P, Chhabra R. Herd and individual animal prevalence of bovine brucellosis with associated risk factors on dairy farms in Haryana and Punjab in India. Trop Anim Health Prod. 2013;45(6):1313–1319. 23354992 10.1007/s11250-013-0362-y

[29] Zheludkov M, Tsirelson L. Reservoirs of Brucella infection in nature. Biology Bulletin. 2010;37:709–715.

[30] Osoro EM, Munyua P, Omulo S, et al. Strong association between human and animal Brucella Seropositivity in a linked study in Kenya, 2012–2013. The American Society of Tropical Medicine and Hygiene. 2015;93(2):224–231. 10.4269/ajtmh.15-0113 PMC4530738 26101275

[31] Tsegay A, Tuli G, Kassa T, et al. Seroprevalence and risk factors of brucellosis in abattoir workers at Debre Zeit and Modjo export abattoir, Central Ethiopia. BMC Infect Dis. 2017;17:1–8. 28125966 10.1186/s12879-017-2208-0 PMC5270313

[32] Mia MM, Hasan M, Pory FS. Occupational exposure to livestock and risk of tuberculosis and brucellosis: A systematic review and meta-analysis. One Health. 2022;15:100432. 36277098 10.1016/j.onehlt.2022.100432 PMC9582573

[33] Zheng R, Xie S, Lu X, et al. A systematic review and meta-analysis of epidemiology and clinical manifestations of Human Brucellosis in China. Biomed Res Int. 2018;2018: 5712920. 29850535 10.1155/2018/5712920 PMC5937618

[34] Nandini P, Jakka P, Murugan S, et al. Immuno-profiling of Brucella proteins for developing improved vaccines and DIVA capable serodiagnostic assays for brucellosis. Front Microbiol. 2023;14:1253349. 37860136 10.3389/fmicb.2023.1253349 PMC10582347

[35] Holt HR, Bedi JS, Kaur P, et al. Epidemiology of brucellosis in cattle and dairy farmers of rural Ludhiana, Punjab. PLoS Negl Trop Dis. 2021;15(3):e0009102. 33735243 10.1371/journal.pntd.0009102 PMC8034737

[36] Ducrotoy MJ, Muñoz PM, Conde-Álvarez R, et al. A systematic review of current immunological tests for the diagnosis of cattle brucellosis. Prev Vet Med. 2018;151:57–72. 29496108 10.1016/j.prevetmed.2018.01.005

[37] Moreno E, Blasco JM, Moriyón I. Facing the human and animal brucellosis conundrums: the forgotten lessons. Microorganisms. 2022;10(5):942. 35630386 10.3390/microorganisms10050942 PMC9144488

[38] Elbers A, Gorgievski M, Zarafshani K, et al. To report or not to report: a psychosocial investigation aimed at improving early detection of avian influenza outbreaks. OIE Revue Scientifique Et Technique. 2010;29(3):435–449. 10.20506/rst.29.3.1988 21309445

[39] Bronner A, Hénaux V, Fortané N, et al. Why do farmers and veterinarians not report all bovine abortions, as requested by the clinical brucellosis surveillance system in France? BMC Vet Res. 2014;10:1–12. 24762103 10.1186/1746-6148-10-93 PMC4036594

[40] Laine CG, Scott HM, Arenas-Gamboa AM. Human brucellosis: widespread information deficiency hinders an understanding of global disease frequency. PLoS Negl Trop Dis. 2022;16(5):e0010404. 35580076 10.1371/journal.pntd.0010404 PMC9113565

