# Supplementary material for: Brucellosis seroprevalence in cattle in China during 2014–2024: a systematic review and meta-analysis
Source: Emerg Microbes Infect. 2024 Oct 25;13(1):2417859. doi: 10.1080/22221751.2024.2417859 (PMC11556282; doi:10.1080/22221751.2024.2417859)
Supplement: Supplement_clean.pdf [file TEMI_A_2417859_SM7338.pdf]

Table S1. The main characteristics of studies included in the study.

First author (Reference)	Score	Province	Survey year	Detection method	Breed	No. Sample	No. Positive
Z.Fan [1]	2	Hunan	2014-2022	RBPT+SAT	unknow	155954	367
Z.Mai [2]	2	Xinjiang	2015	RBPT	unknow	1508	25
			2016			1369	15
D.Xu [3]	2	Shandong	2015	RBPT+SAT	unknow	660	0
			2016			4467	0
Z.Li [4]	5	Guizhou	2015	RBPT+SAT	Dairy cattle	7308	27
			2016			7407	5
			2017			11195	4
H.Li [5]	3	Gansu	2015	RBPT+SAT	unknow	202	0
			2017			87	0
			2018			46	0
			2019			3647	3
			2020			3647	5
S.Liu [6]	4	Xinjiang	2016	RBPT+SAT	unknow	2268	27
			2017			2002	32
X.Chen [7]	4	Anhui	2016	RBPT+SAT	unknow	6056	0
			2017			29097	2
			2018			22726	56
Y.Liu [8]	4	Shandong	2016-2020	RBPT+SAT	Dairy cattle	211452	615
			2016-2020		Beef cattle	34874	0
J.Zou [9]	4	Hunan	2016	RBPT+cELISA	unknow	3845	32
			2017			4661	99
			2018			4063	195
			2019			3496	94
			2020			4180	78
			2021			5862	42
R.Zheng [10]	4	Xinjiang	2016	RBPT+SAT	unknow	1475	35
			2017			1859	72
			2018			1356	47
			2019			2378	69
			2020			2102	39
			2021			1563	20
S.Liu [11]	4	Jiangsu	2017	RBPT+SAT	Dairy cattle	12169	23
			2018			14011	0
			2019			9440	30
			2020			12046	11
X.Yang [12]	4	Xinjiang	2017	RBPT+SAT	unknow	56189	654
			2018			54182	363
			2019			68386	360
J.Zhang [13]	4	Xinjiang	2017	RBPT+SAT	unknow	30303	481
			2018			26894	264
			2019			26771	91
			2020			33088	116
			2021			32685	161

C.Ding [14]	3	Hunan	2017	RBPT+cELISA	unknow	3245	0
S.Yang [15]	5	Shandong	2017	RBPT+SAT	Dairy cattle	42217	4
D.Xu [16]	4	Shandong	2017	RBPT+SAT	unknow	10803	0
Z.Jiang [17]	3	Sichuan	2018	RBPT+SAT	Dairy cattle	764	28
			2019			388	12
			2020			307	0
			2021			384	2
X.Li [18]	5	Qinghai	2018	RBPT+SAT	Dairy cattle	16663	38
			2019			8017	22
			2020			8252	53
			2021			10730	62
			2022			22293	85
			2018		Beef cattle	8019	21
			2019			11448	68
			2020			8696	138
			2021			17455	140
			2022			47642	751
H.Zhang [19]	4	Guizhou	2018	RBPT+SAT	Dairy cattle	1155	5
S.Zhao [20]	5	Henan	2018	RBPT+SAT	Dairy cattle	25088	205
S.Wang [21]	5	Shandong	2019	RBPT+SAT	Dairy cattle	11675	56
P.Gan [22]	2	Jiangxi	2019	RBPT+SAT	unknow	209	13
			2020			270	10
			2021			290	0
Z.Li [23]	4	Guangxi	2019	RBPT+SAT	unknow	4714	0
			2020			5736	0
			2021			6417	0
			2022			7163	8
G.Zhang [24]	4	Xinjiang	2021	RBPT+SAT	unknow	175896	624
X.Gai [25]	2	Shandong	2022	RBPT+cELISA	unknow	989	2
G.Zhang [26]	4	Xinjiang	2022	RBPT+SAT	unknow	167061	1129
W.Zhang [27]	4	Shandong	2023	RBPT+SAT	unknow	9641	3
H.Zhang [28]	4	Xinjiang	2017-2018	RBPT+cELISA	Dairy cattle	1406	95
X. Cao [29]	4	Gansu	2014	RBPT+SAT	Beef cattle	3544	4
					Dairy cattle	1975	2
			2015		Beef cattle	53	0
					Dairy cattle	2282	53
Y. Wang [30]	5	Hubei	2018	iELISA	Dairy cattle	3091	1311
F. Liu [31]	4	Jilin	2015-2017	iElisa	Beef cattle	535	41
F.Li [32]	3	Xinjiang	2017-2018	RBPT+SAT	unknow	11538	396
S.Qin [33]	3	Xinjiang	2019-2020	RBPT+SAT	unknow	987	4

J. Zeng [34]	4	Tibet	2015	RBT+cELISA	Yak	1523	43
J.Zhou [35]	2	Xinjiang	2023	RBPT+cELISA	unknow	446	0
H.Lu [36]	2	Guangdong	2014	RBPT+SAT	unknow	61	0
			2015			117	1
			2016			60	1
S.Chen [37]	2	Guangdong	2014	RBPT+SAT	unknow	61	0
			2015			117	1
			2016			60	1
W.Liu [38]	2	Fujian	2017	RBPT+SAT	unknow	110	0
Q.Yu [39]	5	Gansu	2014	RBPT+SAT	Dairy cattle	79567	206
			2015			72855	426
X.Zhu [40]	3	Shaanxi	2019	RBPT+SAT	Beef cattle	92	0
			2020		Dairy cattle	294	0
F.Pan [41]	3	Guizhou	2020	RBPT+SAT	Beef cattle	165	0
			2021			183	0
			2022			223	0
R.Ren [42]	4	Hainan	2020	RBPT+SAT	Dairy cattle	1690	0
T.Liu [43]	4	Hebei	2015	RBPT+SAT	Dairy cattle	4276	6
S.Zhao [44]	5	Henan	2014	RBPT+SAT	Dairy cattle	23398	428
			2015			10430	181
J.Wan [45]	1	Henan	2015	RBPT	Dairy cattle	218	17
S.Zhao [46]	3	Henan	2022	RBPT+SAT	Dairy cattle	1525	6
					Beef cattle	972	0
Y. Liu [47]	5	Henan	2019	RBPT+SAT	Dairy cattle	12755	283
L.Tang [48]	4	Sichuan	2016	RBPT+SAT	Dairy cattle	650	8
			2017			367	6
			2018			153	0
Z.Xu [49]	4	Jiangsu	2017	RBPT+cELISA	unknow	23475	30
J.Wang [50]	1	Inner Mongolia	2018-2022	RBPT	unknow	159	0
C.Jin [51]	4	Shandong	2018	RBPT+SAT	unknow	19826	9
			2019			10915	1
			2020			11793	0
L.Zhao [52]	5	Qinghai	2018	iELISA+SAT	Yak	1300	6
H.Gou [53]	5	Qinghai	2021	iELISA	Yak	7813	108
H.Song [54]	3	Gansu	2015-2016	RBPT+SAT	Beef cattle	1797	37
					Yak	377	8
					Dairy cattle	5777	110
X.LI [55]	1	Inner Mongolia	2014-2015	iELISA	Dairy cattle	1485	370
X.Li [56]	2	Inner Mongolia	2018	RBPT+SAT	unknow	384	0
			2019			475	3
			2020			471	1
			2021			562	2
			2022			765	0
S.Wu [57]	3	Inner Mongolia	2019	RBPT+SAT	unknow	3292	84
H.Jia [58]	3	Ningxia	2022	RBPT+cELISA	Beef cattle	400	110
X.Ma [59]	4	Ningxia	2021	RBPT	Beef cattle	961	82

					Dairy cattle	1050	355
Xirilamao [60]	2	Qinghai	2018	RBPT	unknow	1360	15
			2019			1209	10
Y.You [61]	2	Hunan	2018	RBPT+cELISA	unknow	60	0
			2019			70	0
			2020			40	0
			2021			80	0
			2022			120	0
Y.Liu [62]	3	Xinjiang	2014	RBPT+SAT	unknow	2053	5
			2015			331	1
G.Gu [63]	3	Shandong	2014	RBPT+SAT	Dairy cattle	161	0
			2015			90	0
			2016			235	0
			2017			339	0
			2018			69	2
Y.Lin [64]	4	Qinghai	2015	RBPT+SAT	Dairy cattle	3270	79
Y.Lin [65]	5	Qinghai	2015	RBPT+SAT	unknow	51977	637
					Dairy cattle	20192	111
					Breeding cow	18951	473
					Beef cattle	12834	53
B.Ding [66]	3	Qinghai	2017	RBPT+SAT	unknow	1494	69
Y.Shao [67]	3	Shandong	2015	RBPT+SAT	unknow	1590	3
L.Sheng [68]	4	Shaanxi	2018	RBPT+SAT+cELISA	Dairy cattle	1227	43
Y.Hu [69]	4	Xinjiang	2014	RBPT+SAT	unknow	2315	104
P.Quan [70]	1	Henan	2018-2019	RBPT+SAT	unknow	60	51
		Inner Mongolia				320	127
X.Ma [71]	4	Xinjiang	2014	RBPT+SAT	unknow	2008	12
			2015			3555	61
			2016			2825	23
			2017			1514	13
			2018			3295	22
			2019			2550	8
X.Wu [72]	4	Xinjiang	2021	RBPT+SAT	unknow	7102	42
F.Zhang [73]	1	Shaanxi	2016	RBPT	unknow	620	0
			2017			691	2
			2018			34	3
			2019			44	1
			2020			94	0
C.Wang [74]	4	Hunan	2017	RBPT+SAT	unknow	4537	2
P.Luo [75]	4	Xinjiang	2014	RBPT+SAT	unknow	21350	664
			2015			19870	818
			2016			18768	409
			2017			18750	523
			2018			15600	320
			2019			12350	222

Q.Yang [76]	2	Xinjiang	2017	RBPT	unknow	1406	96
S.Lv [77]	4	Xinjiang	2019	RBPT+SAT	unknow	4364	64
			2020			2526	29
			2021			1565	11
Z.Shu [78]	3	Xinjiang	2015	RBPT+SAT	unknow	49261	2022
W.Lin [79]	5	Xinjiang	2018	RBPT+SAT	Dairy cattle	153058	37082
			2019			170956	59087
			2020			188305	69610
			2021			201481	82402
			2022			205649	84575
A.Li [80]	5	Xinjiang	2015	RBPT+SAT	Beef cattle	8273	136
			2015	RBPT+SAT	Dairy cattle	4399	12

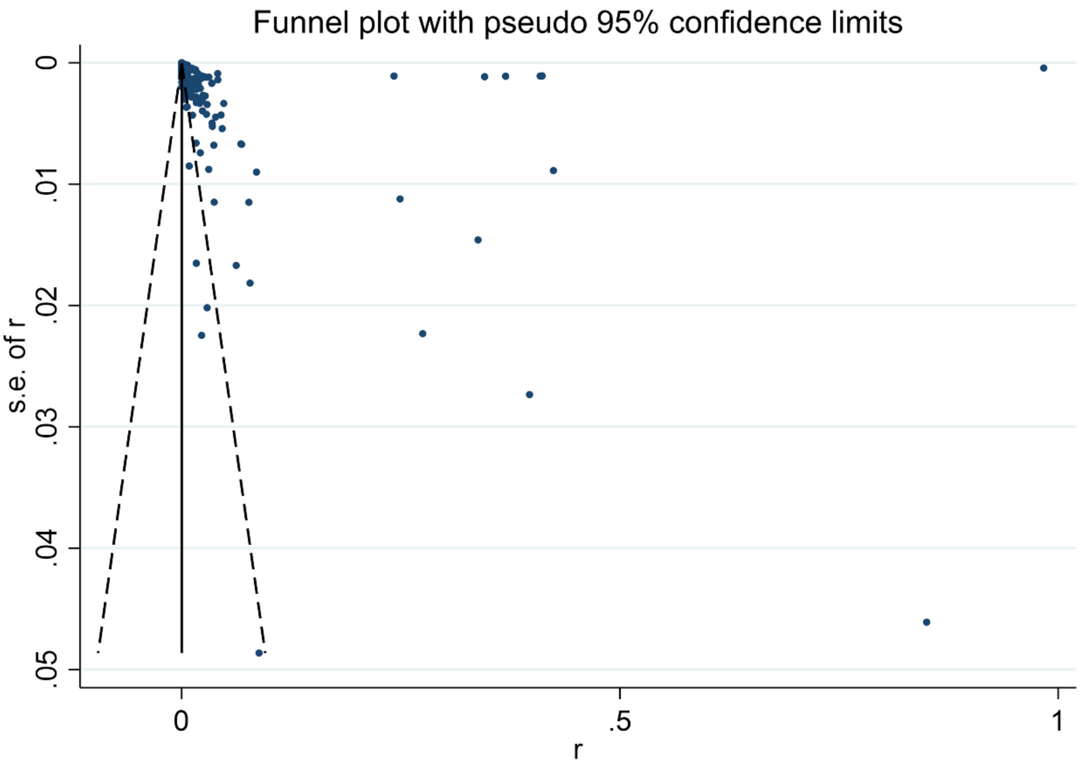

Figure S1. Funnel plot with pseudo 95% confidence limits for assessing publication bias, after Bartlett correction. The plot displays the relationship between the effect size (r) and the standard error (s.e. of r). Each dot represents an individual study. The dashed lines indicate the 95% confidence limits, forming a funnel shape. Symmetry around the central line suggests an absence of publication bias, while asymmetry may indicate its presence. Studies with higher precision cluster near the top, while those with greater standard error scatter toward the bottom.

Table S2. Spearman correlation coefficients (ρ) between average annual cattle population, beef production, milk production, and combined seroprevalence of brucellosis in different provinces of China from 2014 to 2024.

Province	Livestock (AVG.)	Beef production (AVG.)	Milk production (AVG.)	Sero.	Spearman correlation					
					Livestock-Sero.		Beef-Sero.		Milk-Sero.	
					ρ	*p*	ρ	*p*	ρ	*p*
Anhui	114.3	12.1	37.5	0.00%	0.437	0.091	0.228	0.396	0.413	0.112
Shandong	375.2	66.7	265.2	0.00%						
Guangxi	374.9	13.8	10.6	0.00%						
Jiangsu	29.1	3.0	61.0	0.10%						
Guizhou	499.4	20.1	5.2	0.10%						
Gansu	476.0	23.1	56.2	0.40%						
Hunan	428.6	19.7	7.4	0.60%						
Guangdong	158.2	5.1	15.3	0.60%						
Shaanxi	150.0	8.4	118.1	0.60%						
Qinghai	552.0	16.0	33.6	0.90%						
Sichuan	889.6	36.1	67.5	1.20%						
Jiangxi	275.6	14.6	10.1	2.40%						
Henan	562.6	50.1	248.3	2.90%						
Xinjiang	520.9	45.2	191.7	2.90%						
Inner Mongolia	702.7	63.2	683.2	3.30%						
Ningxia	157.2	11.3	219.2	22.10%						
Beijing	12.2	1.0	36.0							
Tianjin	28.1	3.0	56.0							
Hebei	376.6	55.8	469.6							
Shanxi	112.8	7.2	107.5							
Liaoning	305.7	33.4	134.6							
Jilin	374.5	43.3	39.9							
Heilongjiang	502.6	46.4	506.5							
Shanghai	5.7	0.1	30.0							
Zhejiang	14.9	1.4	17.1							
Fujian	43.5	2.5	17.0							
Hubei	278.7	18.0	13.4							
Hainan	60.3	2.2	0.3							
Chongqing	119.0	7.9	4.5							
Yunan	825.4	38.5	62.2							
Tibet	631.7	19.6	41.1							
Hong Kong										
Macao										
Taiwan										

Note: The data were sourced from the Agricultural Production Yearbooks published by the National Bureau of Statistics of China from 2013 to 2023, and we calculated the annual averages. Spearman rank correlation coefficient: $\rho = 1 - \frac{6\sum d_i^2}{n(n^2-1)}$. ρ indicates the strength and direction of the monotonic relationship between two variables. ρ value of 1 indicates a perfect positive correlation, -1 indicates a perfect negative correlation, and 0 indicates no correlation. The p-value represents the probability that the observed correlation occurred by chance under the null hypothesis that there is no true correlation between the variables. If $p < 0.05$, the correlation is considered statistically significant, meaning that at a 95% confidence level, it is likely that a true correlation exists between the variables. Abbreviation: Sero. (seroprevalence)

Table S3. Meta-regression analysis of factors influencing the seroprevalence of bovine Brucellosis in China

Variation	Coef.	Std. Err.	t	P>t	[95% Conf.Interval]
Area	0.020	0.016	1.24	0.218	-0.012, 0.052
Breed	-0.006	0.005	-1.18	0.240	-0.016, 0.004
Survey year	0.006	0.014	0.42	0.672	-0.021, 0.033
Constant	0.015	0.043	0.34	0.736	-0.071, 0.100

Variables such as Area, Breed, and Survey year were analysed, but none showed statistical significance, as indicated by p-values (P>t) greater than 0.05. The 95% confidence intervals (Conf. Interval) for all factors include zero, reinforcing the lack of significant association with seroprevalence. Abbreviations: Coef. (Coefficient), Std. Err. (Standard Error), t (t-statistic), P>t (p-value), and 95% Conf. Interval (95% Confidence Interval).

Table S4. Prediction of the human case numbers based on time series models.

Date	2024		2025		2026		2027	
	Forecast	LCL, UCL	Forecast	LCL, UCL	Forecast	LCL, UCL	Forecast	LCL, UCL
Jan	/	/	4417	2782, 6052	4624	2410, 6837	4810	2227, 7393
Feb	/	/	5691	4055, 7326	5755	3541, 7969	5814	3231, 8398
Mar	/	/	6190	4554, 7826	6198	3984, 8413	6208	3624, 8792
Apr	/	/	7030	5394, 8667	6945	4730, 9159	6871	4287, 9454
May	/	/	7801	6165, 9438	7629	5414, 9844	7478	4894, 10062
Jun	7463	6463, 8463	7320	5447, 9193	7203	4844, 9562	7100	4417, 9782
Jul	8305	6989, 9621	8069	6041, 10098	7868	5409, 10327	7690	4937, 10443
Aug	7843	6338, 9348	7660	5526, 9795	7505	4975, 10035	7368	4565, 10171
Sep	5976	4347, 7606	6004	3795, 8214	6035	3455, 8615	6064	3225, 8902
Oct	4642	3011, 6274	4821	2610, 7032	4985	2404, 7566	5131	2292, 7971
Nov	4704	3071, 6337	4877	2665, 7089	5035	2453, 7617	5175	2335, 8015
Dec	4001	2367, 5635	4254	2041, 6467	4482	1899, 7064	4684	1844, 7525

Note: Abbreviations: LCL (Low Control Limit), UCL (Upper Control Limit).

Table S5. Statistical analysis of fit for ARIMA Model of bovine brucellosis seroprevalence

Model	ARIMA	Model Fit statistics				
		Stationary R-squared	R-squared	RMSE	MAE	MAPE
National-level model	(0,1,4)	.819	.663	.922	54.141	149.603
High-baseline model	(0,1,4)	.728	.619	1.628	54.674	155.237
Low-baseline model	(1,1,1)	.878	.749	.750	137.445	342.599

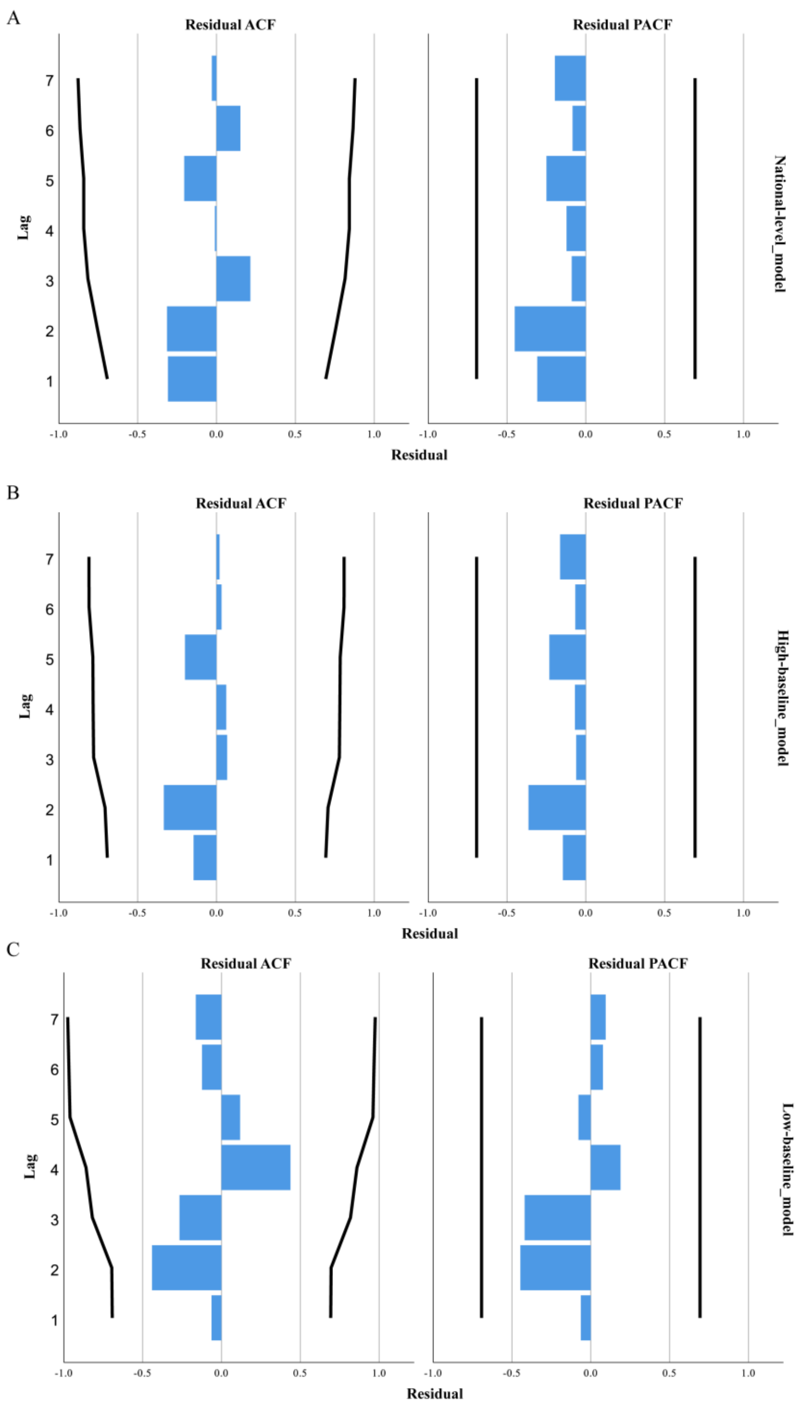

Figure S2. Residual plot of the ARIMA model for bovine brucellosis seroprevalence. The results suggest that all three models exhibit a strong fit, with R-squared values close to 1. This is especially true when it comes to describing the variability of the data, as indicated by the Stationary R-squared values also being close to 1. Nevertheless, the models have elevated RMSE and MAE, highlighting the need for enhancement in predictive precision and revealing substantial forecast discrepancies at certain data points. The mistakes are ascribed to the inadequate amount of data and the existence of outliers in the original dataset. The residual plots of the three models clearly show that both the autocorrelation and partial autocorrelation coefficients are within significant boundaries. This indicates that the residuals follow a white noise pattern.

Table S6. Statistical analysis of fit for ARIMA Model of human cases

Model	ARIMA	Model Fit statistics		Ljung-Box Q(18)			Number of Outliers
		Stationary R-squared	R-squared	Statistics	DF	Sig.	
Human cases model	(1,0,4) (1,0,0)	.924	.924	15.225	15	.435	7

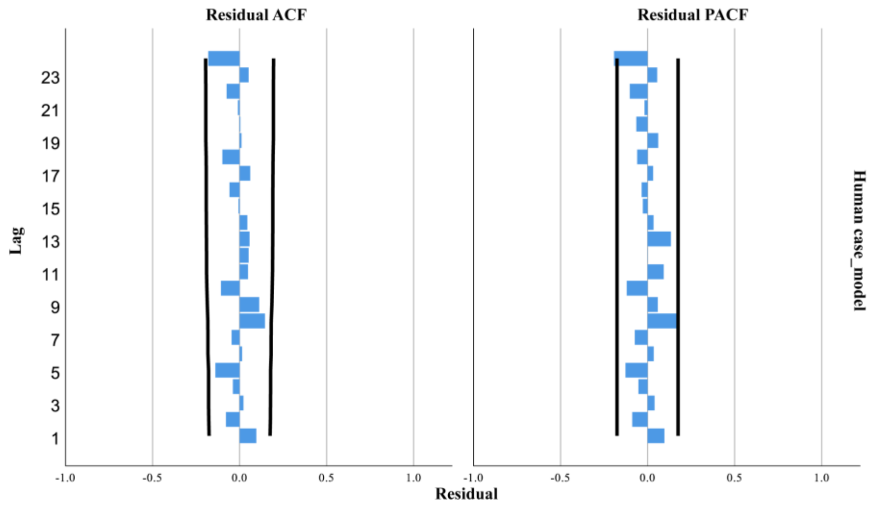

Figure S3. Residual plot of the ARIMA model for human cases. Based on the residual plots, all autocorrelation and partial autocorrelation coefficients for successive lags exhibit no statistically significant variation from zero. The Q-test on residuals resulted in a p-value of 0.435, which indicates that we cannot reject the null hypothesis. This suggests that the residuals constitute a white noise sequence. The time series plot of the actual data and the fitted data exhibit a close overlap, preserving the seasonal impacts of the original sequence. This suggests that the Winter's additive model is a highly accurate fit for the original data.

Reference

[1] Fan Z, Xie Y, Tang X, et al. Statistical Analysis on the Surveillance Results of Livestock Brucellosis in Hunan Provincerom 2014 to 2022. China Animal Health Inspection. 2023;40(08):6-9+72(in Chinese).

[2] Mai Z, Lu Y, Shaya N, et al. Epidemiological investigation on brucellosis of bovine and sheep insome region of Xinjiang in 2015-2016. Journal of Food Safety & Quality. 2018;9(15):4166-4170(in Chinese).

[3] Xu D, Zhuge W, Zhou Y, et al. Monitoring and Analysis of Interstitial Brucellosis in Livestock in Weihai City from 2015 to 2016. Heilongjiang Animal Science and Veterinary Medicine. 2017 (24):115-117(in Chinese).

[4] Li Z, Liu X, Xu C, et al. Sero-epidemiological Investigation on Brucellosis in Cows in Guizhou Province Durinc2015 to 2017. China Animal Health Inspection. 2019;36(04):19-21(in Chinese).

[5] Li H. Investigation and Analysis of Brucella Brucellosis of Cattle and Sheep in ZhangjiachuanCounty from 2015 to 2020. China Cattle Science. 2022;48(02):83-86(in Chinese).

[6] Liu S, Ma L, Yeerjianati T, et al. Epidemiological investigation on Brucellosis of Bull and Sheep in Urumqi County in 2016-2017. Grass Feeding Livestock. 2018 (02):25-33(in Chinese).

[7] Chen X, Zhan H, Hong G, et al. Serological Surveillance of Brucellosis in Cattle and Sheep in Anhui Province during2016 to 2018. China Animal Health Inspection. 2020;37(06):1-4(in Chinese).

[8] Liu Y, Dang A, Zhang D, et al. Analysis on Serological Monitoring Data of Animal Brucellosis in Shandong Province from 2016 to 2020. China Animal Health Inspection. 2021;38(11):7-10(in Chinese).

[9] Zou J, Jiang L, Qu B, et al. Epidemiological Investigation on Brucellosis in Yongzhou City of Hunan Province from 2016 to 2021. China Animal Health Inspection. 2023;40(06):6-11(in Chinese).

[10] Zheng R, Luo P, Shu Z, et al. Serological Surveillance of Brucellosis in Cattle and Sheep in Qinghe County During 2016 to 2021. Grass Feeding Livestock. 2022 (05):39-46(in Chinese).

[11] Liu S, Zhu G, Wang J, et al. Monitoring of Brucellosis in Some Dairy Farms in Xuzhou Area from 2017 to 2020. Animal Husbandry and Veterinary Science and Technology Information. 2021 (07):26-27(in Chinese).
[12] Yang X, Wu Y, Li S, et al. Evaluation on the Purification of Brucellosis in Yizhou District,Hami City of Xinjiangduring 2017 to 2019. China Animal Health Inspection. 2020;37(06):9-11+15(in Chinese).
[13] Zhang J, Luo S. Serological Surveillance on Brucellosis in Cattle and Sheep in Yiwu County of Xinjiang from 2017 to 2021. China Animal Health Inspection. 2022;39(05):7-11(in Chinese).
[14] Ding C, Xiong D, Tan Q. Baseline Survey of Brucellosis in Cattle and Sheep in Loudi City in 2017. Hunan Journal of Animal Science & Veterinary Medicine 2018 (02):25-27(in Chinese).
[15] Yang S, Liang J, Ma Q. Baseline Survey of Brucellosis in Cattle and Sheep in Qingdao City, Shandong Province in 2017. The Chinese Livestock and Poultry Breeding. 2021;17(12):3-5(in Chinese).
[16] Xu D, Zhang H, Yin W, et al. Baseline Survey of Brucellosis in Cattle and Sheep in Weihai City of Shandong Province in 2017. China Animal Health Inspection. 2017;34(10):3-6+14(in Chinese).
[17] Jiang Z, Lu W, Yue J, et al. Analysis on the Monitoring Results of Brucellosis in Bovine and Sheep in JintangCounty,Sichuan Province from 2018 to 2021. China Animal Health Inspection. 2023;40(04):37-40(in Chinese).
[18] Li X, Zhang L, Fu Y, et al. Characterization of Brucellosis Prevalence in Cattle and Sheep Herds in Qinghai Province, 2018-2022. Shandong Journal of Animal Science and Veterinary Medicine. 2023;44(12):14-16(in Chinese).
[19] Zhang H, Fang Y, Xu C, et al. Serological Survey of Brucellosis in Dairy Livestock in Guiyang City in 2018. Guizhou Journal of Animal Husbanday & Veterinary Medicine. 2019;43(04):53-54(in Chinese).
[20] Zhao S, Wang S, Zhao P, et al. Investigation of Bovine Brucellosis Infections in Henan Province in 2018. Chinese Journal of Veterinary Medicine. 2020;56(05):42-44(in Chinese).
[21] Wang S. Epidemiological Investigation of Brucellosis in Cattle and Sheep and Evaluation of Immune Efficacy of S2 Vaccine in Shandong Province in 2019. [Master]: Shandong Agricultural University; 2020.

[22] Gan P, Duan Z, Zeng X, et al. Analysis of Purification Monitoring Results of Brucellosis in Large-scale Cattle and Sheep Farms in Jiangxi Province from 2019 to 2021. Jiangxi Journal of Animal Husbandry & Veterinary Medicine. 2021 (06):38-40(in Chinese).

[23] Li Z, Zhou Y, Huang J, et al. Serological Monitoring of Livestock Brucellosis in Hezhou City,Guangxi ZhuangAutonomous Region from 2019 to 2022. China Animal Health Inspection. 2024;41(02):5-8(in Chinese).

[24] Zhang G, Zhang Z, Yang Y, et al. Analysis and Evaluation on the Quarantine and Purification Effect of Brucellosis in Cattleand Sheep in Hami City in 2021. Grass Feeding Livestock. 2023 (02):27-32(in Chinese).

[25] Gai X, Tian Z, Zhou C, et al. Cross-sectional linvestigation on Brucellosis in Cattle and Sheep in Yantai City,ShandoncProvince in 2022. China Animal Health Inspection. 2022;39(06):53-56+118(in Chinese).

[26] Zhang G, Zhang Z, Yunusi A, et al. Evaluation on the Effect of Prevention and Control of Livestock Brucellosis in Hami City of Xinjiang in 2022. China Animal Health Inspection. 2023;40(05):16-20(in Chinese).

[27] Zhang W, Zheng M, Jiang W, et al. Cross-sectional investigation on Brucellosis in Cattle and Sheep in A City of Shandong Province in 2023. China Animal Health Inspection. 2024;41(02):1-4(in Chinese).

[28] Zhang H, Deng X, Cui B, et al. Abortion and various associated risk factors in dairy cow and sheep in Ili, China. PLoS One. 2020;15(10):e0232568.

[29] Cao X, Li S, Li Z, et al. Enzootic situation and molecular epidemiology of Brucella in livestock from 2011 to 2015 in Qingyang, China. Emerging Microbes & Infections. 2018;7(1):1-8.

[30] Wang Y, Robertson ID, Cheng S, et al. Evaluation of a milk ELISA as an alternative to a serum ELISA in the determination of the prevalence and incidence of brucellosis in dairy herds in Hubei Province, China. Preventive veterinary medicine. 2020;182:105086.

[31] Liu F, Wang D, Yang SC, et al. Prevalence and Risk Factors of Brucellosis, Toxoplasmosis, and Neosporosis Among Yanbian Yellow Cattle in Jilin Province, China. Vector Borne Zoonotic Dis. 2019 Mar;19(3):217-221.

[32] Li F, Wu L, Bayin C, et al. Serological Investigation of Brucellosis and Neosporiasis in Zhaosu County. 2019.

[33] Qin S, Liang J, Tang D, et al. Serological investigation of plague and brucellosis infection in Marmota himalayana plague foci in the Altun Mountains on the Qinghai-Tibet Plateau. Frontiers In Public Health. 2022;10:990218.

[34] Zeng J, Duoji C, Yuan Z, et al. Seroprevalence and risk factors for bovine brucellosis in domestic yaks (Bos grunniens) in Tibet, China. Trop Anim Health Prod. 2017 Oct;49(7):1339-1344(in Chinese).

[35] Zhou J, Wang T, Li Y, et al. Laboratory Investigation on the Prevalence of Major Diseases in Some Large-scale DairFarms in Northern Xinjiang. Modern Animal Husbandry Science & Technology. 2024 (05):14-16(in Chinese).

[36] Lv H. Epidemiological Survey of Brucellosis in Dongguan City and Application of Fluorescence Immunochromatography [Master]: South China Agricultural University; 2018.

[37] Chen S, Zhou Z, Liu X, et al. Serological Antibody Survey of Animal Brucellosis in Dongguan City. Animal Husbandry and Veterinary Science and Technology Information. 2018 (08):12-14(in Chinese).

[38] Liu W. Epidemiological investigation and analysis of brucellosis of cattle and sheep in Shishicity, Fujian province. Journal of Animal Husbandry and Veterinary Medicine Fujian. 2019;41(06):8-10(in Chinese).

[39] Yu Q. Serological Monitoring of Bovine Brucellosis in Gansu Province Over the Past Decade. China Animal Health Inspection. 2016;33(06):4-6+39(in Chinese).

[40] Zhu X, Wang M, Zheng X, et al. Epidemiological Investigation of Livestock Brucellosis in Gaoling District. Journal of Animal Science and Veterinary Medicine. 2019;38(05):42-44(in Chinese).

[41] Pan F, Yao L, Yuan X. Serological Survey and Control Suggestions for Brucellosis in Cengong County, Guizhou Province. Chinese Journal of Traditional Veterinary Science. 2023 (10):13-15(in Chinese).

[42] Ren R. Epidemic Situation of Main Diseases in Two Dairy Farms in Hainan [Master]: Xinjiang Agricultural University; 2021.

[43] Liu T, Xue Z, Li L, et al. Epidemiological Investigation of Bovine Paratuberculosis and Brucellosis in Hebei Province. Heilongjiang Animal Science and Veterinary Medicine. 2016 (05):180-183(in Chinese).

[44] Zhao S, Zhao P, Yan R, et al. Epidemiological Investigation Report of Brucellosis in Large-scale Dairy Farms in Henan Province. Contemporary animal husbandry. 2017 (24):14-15(in Chinese).

[45] Wan J. Investigation of Brucella Infection in Dairy Cows in a Certain Region of Henan Province. China Herbivore Science. 2017;37(03):75-76(in Chinese).
[46] Zhao S, Sheng M, Zhong W. Investigation on Brucellosis infection in the cattle and sheep farmswith large scale in Henan Province. Breeding and Feed. 2024;23(02):17-20(in Chinese).
[47] Liu Y, Ban F, Hu W, et al. Cross-sectional study on the prevalence and risks of spreading brucellosis among cow farms in pingdingshan city of Henan Province. China Animal Health Inspection. 2020;37(12):22-26+38(in Chinese).
[48] Tang L, Yin Y, Zhang W, et al. Investigation of Bovine and Ovine Brucellosis Epidemic in Jianyang City. Today Animal Husbandry and Veterinary Medicine. 2019;46(09):23-24+27(in Chinese).
[49] Xu Z, Xu X, Wang X, et al. Preliminary Investigation on Brucellosis of Cattle and Sheep in Jiangsu Province. China Animal Health Inspection. 2018;35(03):1-5(in Chinese).
[50] Wang J, Yang, Donghui. Analysis of Epidemic Status of Brucellosis in Cattle and Sheep in Baiyun Obo MiningArea in Recent Five Years. Feed Review. 2023 (04):72-75(in Chinese).
[51] Jin C, Jiang W, Guo M, et al. Monitoring and Purification of Brucellosis in Cattle and Sheep in Longkou City. China Animal Industry. 2022 (06):120-121(in Chinese).
[52] Zhao L. Epidemiological Investigation Report of Brucellosis in Yak. Animal Husbandry and Veterinary Science (Electronic Edition). 2021 (01):22-23(in Chinese).
[53] Gou H. Serological Survey Report of Brucellosis in Yaks. Agricultural Family Counselor. 2022 (20):63-65(in Chinese).
[54] Song H. Investigation into the prevalence and Analysis of anti-system effect of brucella brucella in MinLe County [Master]: GanSu Agricultural University; 2018.
[55] Li X, Huo X, Wu Y, et al. Epidemiological Survey, Separation and ldentification of Cow Brucellosis in EasternInner Mongolia. Genomics and Applied Biology. 2017;36(03):921-925(in Chinese).
[56] Li X, Ji Y, Duan L, et al. Baseline Survey Report of Brucellosis-free Areas in Cattle and Sheep in Urad Houqi, Inner Mongolia. Veterinary Orientation. 2023 (04):54-57(in Chinese).

[57] Wu S. Epidemiological investigation and analysis of Brucellosis inUlanqab City of Inner Mongolia [Master]: Inner Mongolia Agricultural University; 2021.

[58] Jia H, Liu J, Wang X, et al. Surveillance and Analysis of Cattle Brucellosis in Yinbei Rural Area of Ningxia. Modern Animal Husbandry Science & Technology. 2024 (04):108-111(in Chinese).

[59] Ma X, He P. Baseline Survey of Bovine and Ovine Brucellosis in Shapotou District, Zhongwei City, Ningxia. Animal Husbandry and Veterinary Science (Electronic Edition). 2022 (12):1-3+24(in Chinese).

[60] Xirilamao. Epidemiological Investigation of Brucellosis in Cattle and Sheep. Veterinary Orientation. 2020 (21):124-125(in Chinese).

[61] You Y, Jia Y, Chen R. Epidemiological Investigation and Control of Brucellosis in Cattle and Sheep. Today Animal Husbandry and Veterinary Medicine. 2023;39(07):14-16(in Chinese).

[62] Liu Y, Huang X, Han M. Serological Epidemiological Investigation of Brucellosis in Cattle and Sheep. Xinjiang Farm Research of Science and Technology. 2017;40(06):29-31(in Chinese).

[63] Gu G, Wang X, Tian Z, et al. Monitoring and Analysis of Brucellosis in Cattle and Sheep in Penglai City. The Chinese Livestock and Poultry Breeding. 2018;14(11):20-22(in Chinese).

[64] Lin Y. Investigation of Dairy Cattle Brucellosis and Tuberculosisin Qinghai Haidong Region [Master]: GanSu Agricultural University; 2018.

[65] Lin Y. Epidemiological Investigation and Analysis of Brucellosis in Cattle and Sheep in Qinghai Province. Shandong Journal of Animal Science and Veterinary Medicine. 2017;38(05):45-46(in Chinese).

[66] Wang Q, Cheng R, Zhao Y, et al. The Analysis and Epidemic Conditions of Three Abortion Related Diseases of Cattle andSheep in Haixi City. Chinese Journal of Veterinary Drug. 2018;52(07):1-7(in Chinese).

[67] Shao Y. Epidemiological Survey of Brucellosis in Cattle and Sheep Scaled Farms in Weihai City of Shandong Province. China Animal Health Inspection. 2016;33(09):5-7(in Chinese).

[68] Sheng L, Zhang L, Dong P, et al. Serological investigation on Cow Brucellosis in jingbian County of Shaanxi Province. China Animal Health Inspection. 2018;35(06):5-7(in Chinese).

[69] Hu Y, Dang X. Analysis of Serological Detection Results of Brucellosis in Humans and Livestock in Tacheng City. Endemic Diseases Bulletin(china). 2016;31(01):67-68+78(in Chinese).

[70] Quan P, Zhang Z, Zhao L, et al. Serological Investigation of Five Diaeasea in Beef Cattle in Some Areas of China. Veterinary Orientation. 2022 (01):24-29(in Chinese).

[71] Ma X, Su Y. Geographic Distribution and Epidemic Regularity of Brucellosis in a District of Urumqi. Xinjiang Farm Research of Science and Technology. 2020 (05):39-43(in Chinese).

[72] Wu X. Epidemiological investigation and prevention measures of bovinebrucellosis in Urumgi [Master]: Xinjiang Agricultural University; 2022.

[73] Zhang F, Yang Y, Du C, et al. Epidemiological Investigation and Analysis of Brucellosis in Cattle and Sheep in Weiyang District, Xi'an City from 2016 to 2020. Breeding and Feed. 2021;20(09):156-158(in Chinese).

[74] Wang C, Xiang D, Tian L, et al. Baseline Survey and Analysis of Brucellosis in Xiangxi Prefecture in 2017. Chinese Abstracts of Animal Husbandry and Veterinary Medicine. 2018;34(06):124-125(in Chinese).

[75] Luo P, Zhang Q. Analysis on Epidemic Characteristics of Brucellosis in Cattle and Sheep in Xinjiang. Grass Feeding Livestock. 2020 (02):49-55(in Chinese).

[76] Yang Q, Deng X, Zhang H, et al. Investigation and Analysis of Brucellosis in Three Pastures of Xinjiang. Progress in Veterinary Medicine. 2021;42(08):126-131(in Chinese).

[77] Lv S, Luo P, Shu Z. Epidemiological investigation of Brucellosis in cattle and sheep in Fuhai County, Xinjiang. Modern Journal of Animal Husbandry and Veterinary Medicine. 2021 (10):84-87(in Chinese).

[78] Shu Z, Zhao W, Wang X. Risk Analysis and Suggestions for Control on the Prevalence and Spread of Cattle and Sheep Brucellosis in Xinjiang. Grass Feeding Livestock. 2015 (06):30-38(in Chinese).

[79] Lin W, Shi G, Dou L, et al. Serological Monitoring of Cow Brucellosis in Shihezi City, 8th Division of Xinjiang Production and Construction Corps. China Animal Health Inspection. 2023;40(10):11-13+20(in Chinese).

[80] Li A, Wang T, Wu X, et al. Investigation on Brucellosis infection of Cattle and Sheep in Circulation Stage in Urumgi City,Xinjianc. China Animal Health Inspection. 2016;33(02):5-8+11(in Chinese).
